# Evaluation of Physical Interaction during Walker-Assisted Gait with the AGoRA Walker: Strategies Based on Virtual Mechanical Stiffness

**DOI:** 10.3390/s21093242

**Published:** 2021-05-07

**Authors:** Sergio D. Sierra M, Marcela Múnera, Thomas Provot, Maxime Bourgain, Carlos A. Cifuentes

**Affiliations:** 1 Department of Biomedical Engineering, Colombian School of Engineering Julio Garavito, Bogotá 111166, Colombia; sergio.sierra@escuelaing.edu.co (S.D.S.M.); marcela.munera@escuelaing.edu.co (M.M.); 2 EPF—Graduate School of Engineering, F-92330 Sceaux, France; thomas.provot@epf.fr (T.P.); maxime.bourgain@epf.fr (M.B.); 3 Arts et Métiers Institute of Technology, Institut de Biomécanique Humaine Georges Charpak, IBHGC, UR 4494, F-75013 Paris; Université Sorbonne Paris Nord, F-93000 Bobigny, France

**Keywords:** physical interaction, smart walker, virtual stiffness, haptic interface, gait analysis, assistive robotics

## Abstract

Smart walkers are commonly used as potential gait assistance devices, to provide physical and cognitive assistance within rehabilitation and clinical scenarios. To understand such rehabilitation processes, several biomechanical studies have been conducted to assess human gait with passive and active walkers. Several sessions were conducted with 11 healthy volunteers to assess three interaction strategies based on passive, low and high mechanical stiffness values on the AGoRA Smart Walker. The trials were carried out in a motion analysis laboratory. Kinematic data were also collected from the smart walker sensory interface. The interaction force between users and the device was recorded. The force required under passive and low stiffness modes was 56.66% and 67.48% smaller than the high stiffness mode, respectively. An increase of 17.03% for the hip range of motion, as well as the highest trunk’s inclination, were obtained under the resistive mode, suggesting a compensating motion to exert a higher impulse force on the device. Kinematic and physical interaction data suggested that the high stiffness mode significantly affected the users’ gait pattern. Results suggested that users compensated their kinematics, tilting their trunk and lower limbs to exert higher impulse forces on the device.

## 1. Introduction

Bipedal walking is one of the most important faculties of humans, as it involves the central nervous system, muscular activation, and the integration of sensory information [1,2]. These systems provide initiation, planning, and execution of gait, responding to motivational and environmental demands of people [3]. Despite the evident gait complexity, individuals usually exhibit smooth, regular, stable, and repeating movements during walking [1].

Recent worldwide estimations report the growing incidence and prevalence rates of health conditions that affect the essential components of gait [4]. There are several neurological conditions, such as cerebrovascular accidents and Spinal Cord Injuries, that are strongly related to locomotion impairments [5]. The effects of these pathologies on human cognitive and physical ability often lead to a total or partial loss of mobility and autonomy [6]. Additionally, the progressive deterioration of cognition and physical ability in the elderly population is directly related to gait disorders [7]. As a result, gait training programs and rehabilitation robotics have become an important research topic for several multidisciplinary teams of health professionals, engineers, and patients [8,9].

According to multiple studies, conventional assistive devices, such as canes, crutches, rollators, manual wheelchairs, ambulatory training machines, and passive orthoses, improve people’s quality of life [10,11]. These solutions have proven to help overcome and compensate for people’s physical limitations by maintaining or improving individuals’ functioning and independence in clinical and everyday scenarios [12,13]. Regarding the walking frames or walkers, these devices improve overall balance by increasing the patient’s base of support, enhancing lateral stability, and providing weight-bearing [14]. In general, walkers exhibit simple mechanical structures and hold excellent rehabilitation potential [10].

In recent years, electronics and robotic technology integration into conventional assistive devices significantly improved and leveraged the functionalities of these devices [15]. For instance, sensing technologies and multiple actuators led to the emergence of wearable robotics, mobile autonomous robots, and robots for gait training and rehabilitation [16]. According to literature evidence, the integration of robotics in gait training and rehabilitation enables several features such as (1) task-oriented and repeatable therapies; (2) intensive activities with programmable difficulty; (3) online follow-up of performance and physiological state of patients; (4) engaging rehabilitation environments through virtual and augmented reality; (5) reliable assessment of patients’ rehabilitation progress; and (6) reduction of the physical effort of therapists [12,17,18]. Technology for gait rehabilitation often comprises robotic wheelchairs, lower-limb exoskeletons, robotic prostheses, robotic rollators, and training devices with body-weight support [8].

In particular, robotic walkers, or Smart Walkers (SWs), are often referred to as a potential tool for gait training, as they integrate simple mechanical structures and multiple sensor interfaces [6]. The SWs overcome issues related to natural balance, users’ energetic costs, the risk for falls, and safety issues, commonly found in conventional walkers and rollators [19]. The main functionalities of SWs include biomechanical monitoring, individuals’ intention estimation, guiding strategies, navigation systems, and fall prevention modules [20,21]. Literature evidence also reports several interfaces for safe and natural interaction with users and the environment [19,20,22].

In addition to the control strategies’ development for an adequate user–SWs interaction, assessments of performance and effects on the patient’s kinematics are also important issues [23,24]. Current tools for gait analysis involve video-based motion capture systems [25], force platforms [26], inertial sensors [27], among others [24]. Additionally, as for robotic walkers, sensors equipped in these devices are also used for gait analysis purposes (e.g., force sensors, laser range-finders, cameras, etc.) [6]. In general terms, recent studies refer to the motion capture systems and force platforms as the gold standard for biomechanical analysis purposes [28,29].

To address gait assistance, smart walkers employ physical interaction strategies such as admittance controllers [30], assistive and resistive forces [31,32], and systems for gait intention detection [33,34]. However, the exploration of kinematics effects of these human-robot interaction strategies (HRI) during walker-assisted gait is still lacking. In particular, there is not enough evidence of the impact on the kinematic chain of HRI strategies based on multiple stiffness levels during walking. Multiple stiffness levels can be used, depending on the patient’s rehabilitation stage, for guiding, gait re-training, and safety provision [30,31,32]. Moreover, high stiffness rendering can be useful for emulating different virtual environments or training conditions.

In this sense, the main contribution of this paper is related to the identification of the biomechanical effects of assistive, resistive, and passive interaction in smart walkers. To this end. this work describes and evaluates three stiffness levels during walker-assisted gait, based on the parameter’s modulation of an admittance controller that virtually resembles a lighter or heavier device. This study also analyzes whether the high stiffness strategy will significantly modify the people’s kinematic using a smart-walker. This work also describes a statistical analysis performed with data collected from a motion capture system and the internal sensors of the AGoRA SW to assess if the effects of such assistance levels were significant [19]. The remainder of this work presents the following sections. Section 2 describes the robotic platform, the interaction strategy, and the experimental setup used in this study. Section 3 presents the primary outcomes related to the physical interaction between the SW and the users, as well as the user’s kinematics. Section 4 presents the discussion about the proposed strategies’ performance and compares related works. This section also outlines the final remarks and future work.

## 2. Materials and Methods

This section describes the robotic platform used during the study and the proposed interaction strategies to provide different assistance levels during gait. This section outlines the experimental setup and the data analysis methods.

### 2.1. Robotic Platform Description

**Figure 1:**
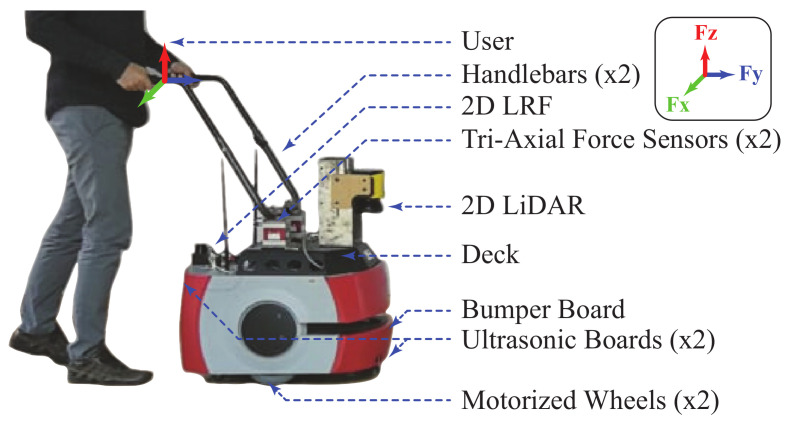
AGoRA Smart Walker illustration, a robotic platform for gait assistance and rehabilitation.

The robot used during this study was the AGoRA Smart Walker. This device is a robotic walker mounted on a commercial robot (Pioneer LX, Omron Adept, Amherst, NH, USA), emulating the structural frame and functionality of an assistive smart walker (See Figure 1).

This platform uses several sensors, actuators, and processing units: (1) Two motorized wheels and two caster wheels for propulsion and stability; (2) two encoders and one Inertial Measurement Unit (IMU) to measure position, orientation, and speed; (3) a 2D Light Detection and Ranging Sensor (LiDAR) (S300 Expert, SICK, Waldkirch, Germany) for environment sensing; (4) two ultrasonic boards for user detection and low-rise obstacles detection; (5) two tri-axial force sensors (MTA400, FUTEK, Irvine, CA, USA) to estimate the user’s navigation commands; and (6) a 2D Laser Range-Finder (LRF) (Hokuyo URG-04LX-UG01, Osaka, Japan) for the user’s gait estimation [19]. The device’s onboard CPU runs a Linux distribution to support the Robotic Operating System (ROS) framework and the software requirements [19]. Moreover, to ensure efficient processing resources, an external computer is used to offload non-critical modules. The platform’s ethernet and WiFi modules allow communication with the external CPU [19].

As previously described by the authors of [19], there is a vertical misalignment between the force sensors on the platform’s deck and the user’s support points on the handlebars (See Figure 1). In particular, the forces along *y-* and *z-axis* read by the sensors will be a combination of the forces along *y-* and *z-axis* at the supporting points. For the sake of simplicity, in this paper, the forces along the *y-axis* were assumed to provide information related to the user’s intention to move. Similarly, this work assumes that the forces along the *z-axis* are a directly proportional estimation of the user’s support on the device. Moreover, according to previous experimental studies, the forces along the *x-axis* are discarded, as they do not provide additional relevant information [19].

### 2.2. Human-Robot Interaction (HRI) Strategy: Case Study

One of the main features of SWs is their ability to respond to the user’s intentions to move compliantly. Notably, several studies report that SWs often equip sensory systems such as force sensors, cameras, joysticks, voice recognition systems, among others, to efficiently detect and extract the users’ navigation commands or intentions to move [6,19,20,21]. The way that these devices respond is controlled by virtually modifying their mechanical stiffness. Specifically, given that users physically interact with SWs by exerting impulse forces and torques, the SWs are often modeled as dynamic systems consisting of a virtual mass connected to a virtual damper (i.e., an admittance controller) [19,30]. With this model, it is possible to generate different velocities on the SW depending on the mechanical stiffness’ values (i.e., virtual mass and damper) and physical interactions with the users. Small stiffness values would lead to easier physical interactions, i.e., the SW resembles a lightweight device, while large values would render a heavier device [19].

In this sense, this study proposes an admittance controller with different stiffness configurations for the AGoRA Smart Walker. This approach aimed to provide different assistance levels to the users, employing multiple dynamic responses on the SW during gait assistance.

**Figure 2:**
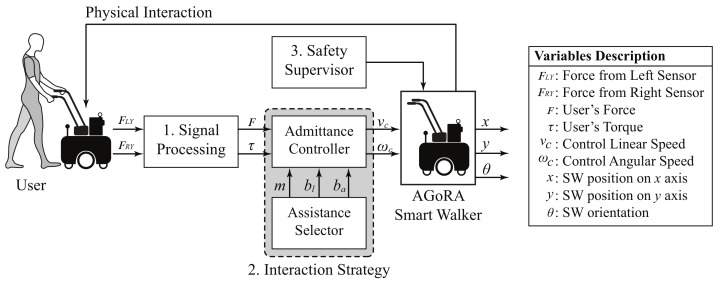
Description of system’s architecture to provide multiple assistance levels.

To this end, the system architecture described in Figure 2 was implemented. The overall architecture is composed of several modules: (1) a signal processing module, capable of filtering raw signals from force sensors and generating the resulting force and torque; (2) an interaction strategy, composed of an admittance controller and an assistance level selector; and finally (3) a safety supervisor, aimed at avoiding hazardous situations for the user (e.g., collisions).

#### 2.2.1. Signal Processing

As previously stated, the forces along the *y-axis* from both sensors provide relevant information about the user’s intention to move. The user’s intention calculation derives from the resulting force and torque exerted on the platform. However, there are some noise issues with these signals. The natural oscillatory pattern of gait often contaminates these force signals [35], and the vibrations associated with the floor can also introduce sources of high-frequency noise [19]. Therefore, these signals require a conditioning and filtering process to remove such components.

This study implements the same filtering process presented by the authors in [19]. In general, the filtering process consists of four steps. The filtering process starts by averaging the signal forces along the *z-axis*, as they contain information related to oscillatory displacements of users’ trunks. Afterward, a band-pass filter removes high-frequency components (i.e., 1 Hz–2 Hz cutoff frequencies). The Weighted Fourier Linear Combiner filter estimates the resulting signal’s cadence [36]. Finally, the cadence is fed to a Fourier Linear Combiner filter to remove oscillatory components from the forces along the *y-axis* from each sensor [37].

The next step estimates the resulting force $\vec{F}$ and torque $\vec{\tau }$, to obtain an indicator of the physical interaction between the smart walker and the user. These final signals were computed using the filtered signals ${\vec{F^{\prime }}}_{LY}$ and ${\vec{F^{\prime }}}_{RY}$ as described in Equations (1) and (2) (*d* is the separation distance between the load cells on the device and is equals to 0.3 m). These calculations did not include the forces along the *z-axis*.

(1) $$\vec{F}={\vec{F^{\prime }}}_{LY}+{\vec{F^{\prime }}}_{RY},$$

(2) $$\vec{\tau }=\left({\vec{F^{\prime }}}_{LY}-{\vec{F^{\prime }}}_{RY}\right)*\frac{d}{2}.$$

In particular, the resulting force $\vec{F}$ was estimated by adding the forces along the *y*-axis on both sensors. It provides information about the users’ intention to start walking. Similarly, the torque $\vec{\tau }$ was estimated using the difference between the force along the *y*-axis and the sensors’ distance. This indicator provides an indirect and partial estimation of the torque exerted on the device and the information about the users’ intention to turn.

#### 2.2.2. Interaction Strategy

Admittance controllers, often implemented in SWs, are dynamic models that enable generating reference velocities from users’ intentions [30]. These controllers allow users to control SWs by exerting forces and torques on the SWs’ handlebars in such a way that they require less effort than if they were to control the robot without the controller enabled [13]. The selection of the controllers’ parameters requires an appropriate tuning process to provide to the users the sensation of easiness and naturalness during physical interaction with the SW [19]. During this tuning process, it is possible to provide different assistance levels by changing the virtual stiffness of the platform [19,30]. This study proposes three assistance levels through three virtual stiffness values. The following sections describe the admittance controller and the assistance selector module.

##### **Admittance Controller** 

In general terms, admittance controllers model SWs as a first-order *mass-damper* system, whose inputs are the force (*F*) and torque (τ) applied to the platform by the user. These controllers output linear (*v*) and angular (ω) velocities. In this manner, two admittance controllers were proposed, as described in Equations (3) and (4):

(3) $$L\left(s\right)=\frac{v(s)}{F(s)}=\frac{\frac{1}{m}}{s+\frac{b_{l}}{m}},$$

(4) $$A\left(s\right)=\frac{\omega (s)}{\tau (s)}=\frac{\frac{1}{J}}{s+\frac{b_{a}}{J}},$$

where *m* is the walker’s virtual mass, *J* is the virtual moment of inertia of the walker, and $b_{l}$ and $b_{a}$ are damping constants. These equations describe the transfer function of each controller. L(s) stands for Linear System, and A(s) stands for Angular System.

##### **Assistance Selector** 

The values of the controllers’ parameters determine the mechanical stiffness of the SW. Thus, by changing the virtual mass, inertia, and damping constants, it is possible to provide multiple assistance levels. In this work, the term “*assistive level*” refers to a stiffness configuration that provides an easy interaction and requires low physical efforts. In contrast, the term “*resistive level*” refers to a stiffness configuration with which the device opposes the users’ intentions to move. Three stiffness levels designed for this work provide the following assistance levels:

- **Assistive Mode (AM):** This configuration aimed to provide the easiest and lightest behavior on the SW. Given that the AGoRA Walker is mounted on a heavy robotic platform (i.e., 70.2 kg), low mass and inertia values were required. Moreover, to ensure stability and balance during walking, the inertia value was designed to be at least twice the virtual mass. By means of several experimental tests, the following values were used: m=0.5 kg, $b_{l}\;=\;4\;$ N·s/m, J = 2.1 kg·m^2^/rad and $b_{a}\;=\;2\;$ N·m·s/rad.
- **Resistive Mode (RM):** This configuration aimed to make the SW oppose the users’ intentions. With this mode, the device was heavier and more difficult to maneuver by users. Given that this study assumes that people with higher Body Mass Index (BMI) values could exert higher force and torque values on the device, a unique stiffness configuration was not suitable. This mode’s virtual mass was at least ten times greater than the virtual mass of the AM. The value of the virtual inertia remained unchanged. The following values were used: m=10 kg, $b_{l}\;=\;\beta \;$ N·s/m, J=2.1 kg·m^2^/rad and $b_{a}$= 7 N·m·s/rad. The calculation of the damping constant of the linear system (β) employed the subjects’ weight, as follows:
  (5) β=0.375·weight−12.5.
  The values of the model presented in Equation (5) were estimated empirically, in such a way that a subject with a maximum weight of 120 kg or a minimum weight of 55 kg could move the device with moderate resistance. Five healthy subjects that did not participate in this study participated in several trials to determine this model. The subjects’ task was to freely interact with the smart walker with the proposed model, which validated the resistive behavior achieved with these constants.
- **Passive Mode (PM):** This configuration disabled the admittance controllers and the device’s brakes. Thus, the walker worked as a conventional wheeled walker with this mode.

#### 2.2.3. Safety Supervisor

As presented by the authors in previous works, the AGoRA Smart Walker includes several safety rules that constraint the walker’s movement when hazardous situations are detected [19].

On the one hand, the device movement is only allowed if the user supports itself on the walker handlebars and stands behind it. Moreover, using the information gathered from the 2D LiDAR mounted on the device, the walker’s speed is constrained when surrounding obstacles are detected. Similarly, to avoid situations that could lead to falls, the device cannot rotate on its axis or move backward. Finally, an emergency button is placed on the platform’s deck to disable the motors if required.

On the other hand, an external CPU is monitoring the AGoRA Smart Walker’s systems’ behavior. If the operator determines that the device is malfunctioning, he can remotely disable it. The supervisor’s safety restrictions are redundant, executed from the onboard computer and the external computer. In case of communication loss with the external computer, the device can continue running the safety supervisor autonomously.

### 2.3. Experimental Protocol

This section describes the implemented experimental protocol to assess the physical interaction between the users and the AGoRA Smart Walker and the users’ kinematics during the proposed interaction strategies.

#### 2.3.1. Session Environment

This study took place at the Motion Capture and Analysis laboratory of the Department of Biomedical Engineering at the Colombian School of Engineering Julio Garavito, Bogotá, Colombia. This laboratory counts with 7 VICON (Oxford, UK) cameras operating at 120 Hz. Before the experiments, the researchers calibrated the motion analysis system with the VICON active wand. The cameras were able to capture an area of 6 × 6 m^2^ (See an illustration of the VICON set up in the following link: https://1drv.ms/u/s!Anv-5biOmdEOhYs4FD4uqptnom1qsA?e=zJdzUJ, accessed on 17 February 2021). A private room was set aside for the preparation of participants before each session.

#### 2.3.2. Participants Recruitment

The university’s ethics committee approved this study, certifying that it agrees with Helsinki’s declaration. This study is also part of the *Development of an Adaptable Robotic Platform for Gait Assistance and Rehabilitation (AGoRA)* Project. The inclusion criteria were as follows: Adults over 18 years old, height between 1.60 m and 1.90 m, weight between 50 kg and 110 kg, and ability to read and sign the informed consent form. The exclusion criteria were as follows: any physical or neurological conditions and subjects with gait assistance requirements.

A group of 11 healthy male volunteers accepted to participate in the experimental trials. The researchers formally recruited all the participants, which provided their signed written consent to participate in the study. The participants were randomly recruited at the facilities of the Colombian School of Engineering Julio Garavito. Moreover, none of the participants had prior experience using the AGoRA Smart walker to avoid learning of the assistance levels. The subjects did not report any history of injuries or musculoskeletal dysfunctions. Table 1 summarizes the anthropometric measures of the participants.

#### 2.3.3. Session Procedure

The required clothing for the trials was only tight-fitting lycra shorts. At the beginning of the session, the researchers instructed the participants on the session’s procedures and the study objectives. Moreover, the participants provided their signed informed consent, and the researchers solved any additional questions. The researchers asked the participants to change their clothes into the clothing mentioned above. Participants were to be barefoot and without any extra clothing or accessories.

**Figure 3:**
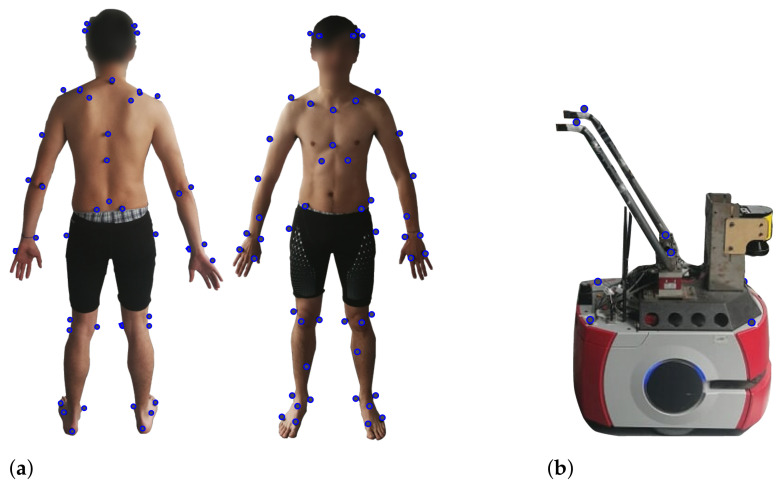
(**a**) Markers’ setup on subject. (**b**) Markers’ setup on the SW.

As described in Figure 3a, the researchers fitted the subjects with 64 reflecting markers (14 mm diameter), according to the full-body setup described by [38]. Similarly, eight markers were placed on the SW, using the setup shown in Figure 3b.

**Figure 4:**
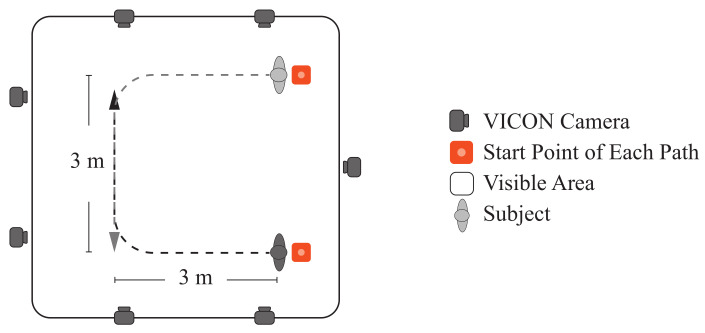
Reference paths for the experimental trials in the motion analysis laboratory. The area that the cameras were able to capture was 6×6 m^2^.

Each session was composed of four parts corresponding to the three assistance levels and an additional trial without the SW (i.e., referred to as Unassisted Mode (UM)). During each session’s part, the users followed one path with turnings in two directions at their preferred speed (See Figure 4). The users had to perform three repetitions in each path’s way, i.e., six repetitions per assistance mode. There was no path marking on the floor, only reference marks at the beginning, the turning points, and the route’s end. The turning radius was approximately 0.8 m. Before recording each mode’s data, the users had a training period to understand the assistance level to be assessed.

Before each recording, the motion analysis system and the AGoRA Smart Walker internal systems were synchronized by gently hitting the force sensors with a stick, while the cameras captured its motion. To this end, the synchronization stick required an additional marker on top of it. Given the uncertainty at measuring the exact impact on the force sensors, with this method, the maximal difference between the two systems was equal to the difference between two consecutive frames (i.e., around 8 ms).

There were no breaks between trials. However, when switching between the different operation modes (i.e., assistance levels), a short period of 3 min was required. During this period, the researchers performed software adjustments to switch the assistance level and review the stored data’s quality. Moreover, the users only interacted with the Smart Walker during the execution of the path. A researcher moved the Smart Walker from the end of the last route to the beginning of the next one.

After each trial, a researcher stored software logs in the VICON Workstation and the AGoRA Smart Walker’s external CPU. The software Nexus 2 (VICON, Oxford, UK) [39] allowed the storage of VICON data and ROS bags stored walker’s data [40]. Each subject was only required to participate in one session.

#### 2.3.4. Outcome Measures and Data Analysis

The Nexus 2 software allowed biomechanical data curation and pre-processing. Then an inverse kinematics approach, using the full-body model reported by [38], was executed with a multi-body kinematics optimization algorithm OpenSim software [41]. The Matlab R2018a Software and the Biomechanical ToolKit Library (BTK) allowed the computation of interaction and kinematic indicators [42,43]. Finally, a resulting outcome was obtained by averaging the kinematic and interaction indicators from the six repetitions (i.e., three repetitions in each direction of the path) for each subject and each assistance mode.

This study proposes several quantitative indicators to measure the users’ performance during trials and the effects of the assistance levels on the physical interaction between users and the AGoRA Smart Walker. Among these, this work estimates gait’s spatiotemporal parameters such as speed, cadence, cycle duration, and the number of cycles. Furthermore, the inverse kinematics outcomes allowed the estimation of the average range of motion (ROM) for hip, knee, and ankle joints in the sagittal plane were estimated. These indicators were calculated for each gait cycle, using the knee flexion angle curve to detect each cycle’s beginning and end. The SW’s sensory interface also recorded the resulting force and torque exerted by users on the device. The estimation of all indicators required each trial’s complete information without separating the straight parts or curves. The signal processing cropped the data from the users’ first step to one step before reaching the end of the path. Table 2 presents a brief explanation of these descriptors.

Regarding statistical analyses, the significance level for all tests was set to 0.05. Moreover, descriptive statistics were used to report the outcomes of the study. All values were reported using the mean plus or minus standard deviation. To determine the distributions of the collected information, Shapiro-Wilk normality tests were carried out. To assess the existence of statistically significant differences, two types of tests were performed. In the case of parametric data, one-way analysis of variance (ANOVA) for repeated measured was conducted. In contrast, Friedman tests were conducted for non-parametric data. Finally, two types of posthoc tests were performed. In the case of parametric data, Bonferroni posthoc tests were performed. In contrast, Conover posthoc tests with Bonferroni correction were used for non-parametric data. All the statistical tests were performed using R 3.6.2 and RStudio Desktop software [44].

## 3. Results

A total of 264 trials divided into 11 sessions (i.e., one session per subject) were performed and successfully recorded. This section describes and illustrates the outcomes of the study.

### 3.1. Physical Interaction Results

The information provided by the tri-axial force sensors on the platform’s deck was used to estimate the physical interaction between the smart walker and the users. In this sense, Table 3 summarizes the values of resulting force and torque values for each assistance level, where AM stands for Assistance Mode, PM stands for Passive Mode, and RM stands for Resistance Mode. Moreover, peak values for these signals are also reported. In this case, no data were reported for the unassisted trials (i.e., without the SW).

Considering that significant differences were found for some parameters, pairwise comparisons were performed. Table 4 summarizes the results of the posthoc tests.

**Figure 5:**
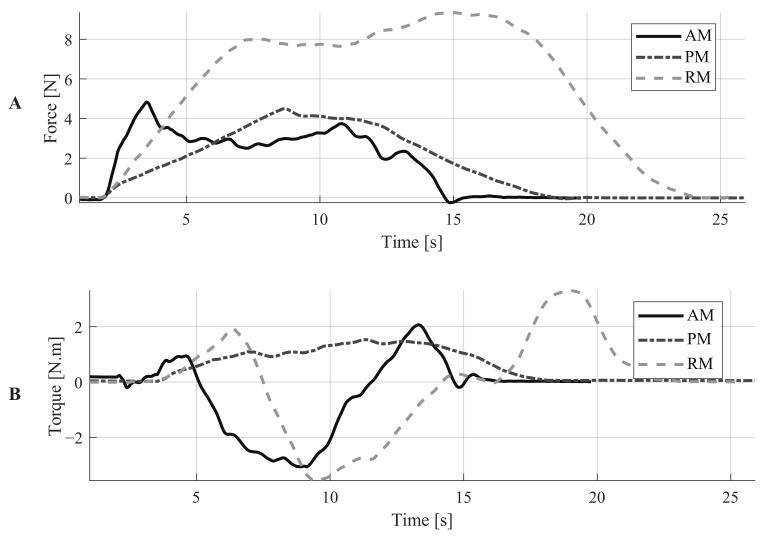
Illustration of force and torque signals for one subject: Assistance Mode (AM), Passive Mode (PM), Resistance Mode (RM).

In addition to the above, to illustrate the behavior of the resulting force and torque signals’ behavior under each assistance level, Figure 5 shows the recorded signals for one representative subject. Particularly, no significant differences were found for the force and torque signals within the same assistance mode (The *p*-value obtained for the force signals was 0.173, and the *p*-value obtained for the torque signals was 0.7168).

### 3.2. Kinematic and Additional Results

Several indicators of the users’ performance were estimated by employing the data collected by the motion capture system. On the one hand, to characterize users’ gait, several parameters such as gait speed, cadence, average gait cycle duration, and the number of gait cycles were calculated. On the one hand, to assess the effects of the assistance levels on the lower-limb kinematic chain of users, the range of motion (ROM) was estimated for ankle, knee, and hip joints. Likewise, the trunk’s angle was also estimated using the marker located at C7 (i.e., at the 7th Cervical Vertebra) and the two markers located at the posterior of the iliac crest. Moreover, data related to trials’ mean duration were also calculated. A summary of this information is reported in Table 5, where the robotic platform’s linear and angular speed are also reported. In this table, UM stands for Unassisted Mode, referring to the conducted trials without the smart walker.

Significant differences were found for all the reported parameters. In this sense, pairwise comparisons were performed using Conover or Bonferroni posthoc tests. Table 6 shows the results obtained for such tests.

**Figure 6:**
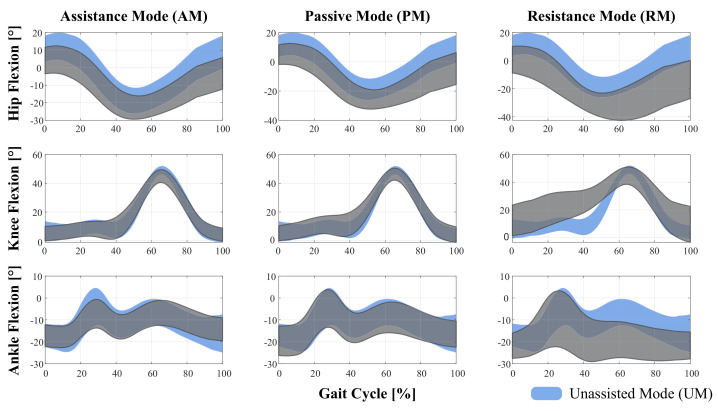
Comparison of sagittal plane joint angles for the assistance levels. Each graph was generated using average gait cycles and standard deviations within mode.

An illustration of the hip, knee, and ankle joints’ behavior is shown in Figure 6, where the average gait cycle is presented for each assistance level and joint. The average gait cycle for the unassisted mode (UM) is also presented used for comparison purposes.

## 4. Discussion

All the subjects succeeded during trials, and no cases of misunderstanding of the AGoRA Smart Walker behavior were reported. In this work, the sample size is considered small; however, studies with walkers have been conducted with similar sample sizes [14]. On the one hand, regarding the interaction strategies, the users were entirely in control of the walker’s movements in all of them. For the assistive (AM) and resistive (RM) modes, an admittance controller was used to generate angular and linear speeds by taking the force and torque exerted by the users on the device. For the passive mode (PM), the walker’s speed controller and brakes were disabled, and it was meant to be operated as a conventional four-wheeled walker. Thus, under the PM, the user only had to push the device to make it move. Moreover, in terms of users’ safety, the supervisor was only active for the AM and RM, given that it was designed to override the speed controller when hazardous situations are detected. Thus, although the safety supervisor was disabled for the PM, no collisions occurred during trials.

On the other hand, according to the results presented in the previous section, several effects on the physical interaction and kinematics of the users were obtained. Notably, changes in the virtual stiffness programmed in the device (i.e., assistance level) significantly impact users’ performance during trials. Moreover, despite the limited test area (i.e., paths’ area of 3 × 3 m^2^), it was sufficient to attain a stable gait. As reported in the literature, between 3.5 and 5.2 steps are required to achieve a steady-state gait [45]. According to our results, the average number of walking cycles (i.e., two consecutive steps) was around 6.7 to 9.3 cycles, ensuring enough space for the users’ gait to stabilize. Besides, the turning radius in the curves was wide enough to prevent users from having to reduce their speed.

### 4.1. Physical Interaction Results

Regarding the physical interaction between the users and the SW, four indicators were measured. On the one hand, the user’s mean force exerted was estimated during trials for each assistance mode. As reported in Table 3, more significant efforts were required from the user to handle the device during the resistive mode (RM). In contrast, the assistive mode (AM) allowed the most effortless interaction, as the mean exerted force by the users was lower than in passive mode (PM) and RM. This outcome indicated that the proposed impedance configurations permitted to have the expected behavior for assisting the subject. Moreover, by analyzing the posthoc tests results presented in Table 4, it can be seen that this indicator exhibited significant differences for all the pairwise comparisons (p<0.05). These results might suggest that the proposed assistance levels allowed us to provide completely different dynamic responses on the AGoRA Smart Walker. In the same manner, the peak force values exhibited similar behavior. This indicator measures the initial contact between the user and the SW, and it estimates how difficult it is to start walking with the device. In this case, the highest value was also registered for the RM and the lowest value was obtained with the AM.

An illustration of these parameters can be found in Figure 5A, where the force signals of a representative subject are shown. These signals describe an interesting outcome related to the initial force required to move the device. Comparing the AM and the PM, the peak force is higher with the AM. However, the required force to keep the device moving is lower for the AM than for the PM. The admittance controller explains this outcome as it models the walker as a dynamic mass-damping system. In this way, it is possible to make the smart walker feel more lightweight with the virtual mass. Moreover, the damper prevents the propagation of the natural oscillations of gait to the walker [19,46]. The combined effect of these elements induces inertia that must be overcome by the user to start walking. However, these elements also ensure that the walker’s movement can be maintained with less effort compared to the PM. In a real application, this behavior makes the AGoRA walker suitable for providing stability to users and assisting users’ gait without requiring as much effort as in the PM.

On the other hand, the mean and peak torque values were also estimated. Regarding the mean torque, significant differences were found for all the assistance levels. Moreover, by analyzing the magnitudes of this parameter, the highest mean torque values were obtained with the RM and the lowest ones with the PM. These results confirm the fact that the SW was virtually more challenging to handle with RM. Regarding the peak torque parameter, similar results were obtained. Significant differences were found for all the assistance levels. The highest values were obtained with the RM and the lowest ones with PM. Moreover, it can also be noted that the standard deviation values were higher with the RM, for both mean and peak torque parameters. This suggests that how users maneuvered the SW was more variable with this level of assistance.

Similar studies to this work have been reported by [30,31,47]. Specifically, ref. [47] implemented several motion control algorithms based on impulse force information. Moreover, this study evaluated the comfortableness of such control strategies. This study concluded that users prefer to interact with devices that provide good maneuverability, require small forces to move, and ensure safety [47]. Although this study analyzed multiple sets of control parameters, no resistive mode was evaluated. Moreover, no kinematic information was reported. Regarding the work presented by [31], the effects of multiple assistive and resistive forces were addressed. The authors reported using foot-switches for gait analysis purposes, finding that cadence, stride length, and double support phase were affected [31]. This study did not measure the interaction force with users, and thus the movement of the device was not based on users’ intention (i.e., which might lead to hazardous situations). Moreover, applying constant assistive or resistive forces could hinder the implementation in real scenarios. Finally, a dynamic modulation strategy for an admittance controller was proposed by [30]. In this study, the parameters of the controller were modified to guide users through the desired path. Although this controller is suitable for assisted navigation purposes, the effects on users’ kinematics were not reported.

### 4.2. Kinematic and Additional Results

Using the information captured by the motion analysis system, several indicators of users’ lower-limb kinematics were estimated (See Table 5).

On the one hand, the users’ and SW’s speeds were registered for all the assistance levels. Regarding the users’ speed, the RM induced the lowest values (0.345±0.043 m/s), mainly because with this configuration the SW opposed the users’ intentions to move. The highest speed values (0.774±0.016 m/s) were reached under the unassisted mode (UM). Particularly, the users reduced their walking speed to 57.56%, 59.57%, and 44.57% of their unassisted speed, with the AM, PM, and RM, respectively. Moreover, statistical tests suggested significant differences for all pairwise comparisons of the users’ speed (See Table 6). This outcome suggests that each assistance level could provide a completely different walking behavior of users. Furthermore, the gait speeds obtained in this work are slightly different from those from the literature evidence. Particularly, several studies report walking speeds ranging from 0.9 to 1.25 m/s [28,31]. Thus, the AGoRA Smart Walker allows slower speeds than the average unassisted walking speed [48]. However, the AGoRA Walker is a rehabilitation device aimed at being used in clinical scenarios, where medical staff often require slower and controlled speeds to correct inappropriate gait patterns [48].

Regarding the SW motion, the linear speed showed similar behavior to users’ speed, where the lowest values were obtained with the RM (See Table 5). This parameter also exhibited significant differences between all the assistance levels. The SW’s angular speed exhibited similar behavior. Significant differences were obtained for all pairwise comparisons (See Table 6). These outcomes also support the fact that each assistance level provides completely different walking behaviors for the users.

On the other hand, gait parameters such as cadence, cycle duration, and the number of cycles were also calculated. The differences between all the assistance levels were found to be statistically significant for these parameters (See Table 6). Regarding cadence, literature evidence suggests that the users’ cadence in this study was nearly 50% of the average cadence during unassisted walking in healthy adults [49]. This discrepancy might be supported by the fact that this work’s experimental environment was considerably smaller to reach average gait speeds, and thus average cadences [31]. The lowest cadence was obtained with the RM, and this outcome is supported by the most prolonged gait cycles also obtained with the RM (See Table 5). The RM induced more step cycles, thus it can be deduced that shorter steps were obtained under the RM.

In terms of the effects of the assistance levels on the lower-limb kinematics chain, the range of motion (ROM) was calculated for hip, knee, and ankle joints in the sagittal plane (See Table 5). Moreover, a comparison of such flexion angles with the unassisted mode is shown in Figure 6. Regarding hip joint, significant differences were found for nearly all pairwise comparisons (See Table 6). Particularly, no differences were found for AM vs. PM and PM vs. UM comparisons. Reductions of 8.41%, and 0.87% of the unassisted ROM were obtained for the AM and PM, respectively. However, an increase of 17.03% was obtained for the RM. This result might suggest that when the device opposes the users’ movement intention, they compensate their motion with slower and wider walking patterns, to exert a higher impulse force (i.e., higher forces along the *y-axis*, see Table 3). Furthermore, by analyzing the hip flexion curves presented in Figure 6, it can be noted that for all assistance levels a reduction of the initial flexion angle occurred in comparison to the UM. Such reduction could be caused by compensation on the trunk inclination angle during walker-assisted gait (See Figure 6).

Regarding the knee joint, the ROM was smaller than the UM for all the assistance levels provided by the SW. As described in Table 6, significant differences were found between each assistance mode and UM, whilst no significant differences were found for AM vs. PM. Regarding the ankle joint, the PM and RM induced increases in the range of motion of this joint (See Table 5). This result also supports the fact that, when users interact with the SW, they tilt the trunk and lower limbs to generate greater impulse forces on the device (See Figure 6). In this sense, it can be noted that the ankle flexion was higher with the RM (34.9±9.8°). These results suggest that RM is the level of assistance that most affected the users’ gait pattern.

In addition to the above, to better understand the behavior of users’ trunk kinematics, the trunk’s angle was estimated along the sagittal plane for all the assistance levels. Specifically, this angle was calculated using the ground plane as a reference, so that a person completely standing would have a 90° angle. Significant differences were found between the assistance levels. Regarding the outcomes of pairwise comparisons, significant differences were found between all modes compared to the RM. Moreover, increases of 17.32%, 19.74%, 16.48% in trunk’s angle were found for AM, PM, and UM, compared to the RM. This outcome suggests that users tilted the most during the resistive behavior of the Smart Walker.

An additional parameter related to the trial duration was also calculated. As expected, the RM’s trials were the longest ones, and the UM trials were the shortest ones (See Table 5). Moreover, significant differences were found for all pairwise comparisons, supporting the fact that each assistance level provides a completely different interaction with the SW (See Table 6).

In terms of safety provision during each of the assistance levels, several aspects are worth mentioning. In particular, Pervez et al., proposed a danger index to estimate user’s safety during mobility assistance [50]. Even though this study does not calculate such indicators, it avoids most of the unsafe situations highlighted in Pervez et al., On the one hand, it is pointed out that for safe assistance there should not be any appreciable speed mismatch between the assistive robot and the user. All the assistance levels proposed in this work guarantee that the smart walker moves at the desired speed of the user. Even in the RM when the smart walker is harder to push, the speed of the user is adjusted to a slower velocity that matches the smart walker’s speed. Regarding obstacles management, the danger index formulation suggests that the robot should not be very close to obstacles nor collide with them. In this case, the safety supervisor maintains the smart walker away from obstacles, as it limits the speed of the walker when approaching an obstacle. This safety condition is not guaranteed in the passive mode, as the brakes and motors are completely disabled. Additionally, the danger index also considers vibrations and jerks. In these cases, the AGoRA smart walker uses an admittance controller that acts as a low pass filter, mitigating any vibrations or jerks.

Finally, although this work was performed on healthy subjects, it is expected that, when testing the assist levels on patients with gait limitations, similar behaviors in kinematic and physical parameters will be obtained. This means that, regardless of the gait condition, the RM will be harder to interact with, and thus patients will exhibit slower gait patterns and increased interaction forces. Moreover, as previously reported in the literature, it is expected that older adult patients or patients with gait dysfunctions will present slower gait patterns in all modes, and will have the less muscular capacity to exert forces on the device [51,52,53].

### 4.3. Final Remarks and Future Works

One of the main findings of this study is related to the kinematic and interaction parameters during the resistive mode (RM). Although the RM opposes the user’s intention to move, it might induce muscular training during rehabilitation processes; the level of resistance could be configured to meet each user’s specific needs. This assistance level also induces slower gait patterns compared to the reported studies in the literature. However, these could be interpreted as a safety strategy, where slower gait patterns might help users to avoid collisions and stumbling during walking. Additionally, the force data gathered during the RM provided insights into possible applications of muscular training.

This study was carried out employing the AGoRA Smart Walker, which is mounted on a commercial robotic platform. This selection was made as the Human–Robot Interface of this assistive device was validated in a previous study with healthy subjects [19]. Moreover, this platform can provide enough safety constraints to guarantee users natural and proper interaction.

One of the limitations of this study is that it lacks EMG information that supports these findings related to physical interaction under the RM. Moreover, it should also be noted that a limitation of this research is the sample size and the participation of only healthy subjects. However, this study is the first approach to the use of controllers with different levels of assistance in walker-assisted gait, and further analyses are required in populations with gait problems, such as older adults or patients with neurological conditions.

Regarding the behavior of the assistive (AM) and the passive modes (PM), they may be useful in patients with lower assistance requirements, as the device allows faster and less controlled movements. However, considering that, during the PM, no speed controller is active, hazardous situations might occur as neither safety supervisor is active. During this mode, the user is completely in control of the smart walker motion.

By comparing the AM and PM behavior, AM can be beneficial to users as it allows the dynamics of the device to be removed. Specifically, the AGoRA Walker is a device mounted on a heavy commercial robotic platform. Thus the implementation of the admittance controller facilitates the user’s interaction with the device. Specifically, the smart walker can be configured to render a lightweight (i.e., as the AM) or bulky device (i.e., as the RM) by setting small or large values on the virtual mass parameter of the admittance controller. It has been reported that these modifications could be suitable to assist activities of daily living, like walking up and down on-ramps using a smart walker [13]. To validate the performance, safety, and effects of the interaction strategies, this study was conducted on a group of healthy subjects, before their deployment in a clinical setting. Moreover, as reported by several related works, the implementation of assistive and resistive strategies might be useful to provide guidance and gait re-training in clinical scenarios [30,31,32]. In particular, the proposed strategies in this work can be easily adapted to cognitive interaction strategies (e.g., assisted navigation) where the smart walker modifies its virtual stiffness to provide feedback in path following, guidance, and navigation tasks.

In addition to the above, it is worth mentioning that the paradigm of multiple assistance levels is not exclusive to walker-assisted gait. In particular, these strategies can be easily implemented in other assistive devices such as exoskeletons or hybrid devices [54,55,56]. Finally, future works will address the assessment of these interaction strategies in a clinical scenario with pathological patients or older people. In this work, the RM was configured according to the weight of the users. Therefore, future works will also address the development of a multivariate model to determine the correlation between the users’ weight and the ability to maneuver the SW. Future studies will also assess more complex walking tasks, including longer and more challenging paths and tasks.

## Figures and Tables

**Table 1:** Summary of anthropometric measures of the volunteers that participated in the study.

Subject	Age [y.o.]	Height [m]	Weight [kg]	Body Mass Index (BMI)
1	23	1.80	72	22.20
2	26	1.79	70	21.80
3	28	1.79	90	28.10
4	20	1.87	95	27.20
5	23	1.78	72	22.70
6	24	1.76	62	20.00
7	23	1.62	58	22.10
8	23	1.79	90	28.10
9	22	1.68	60	21.30
10	23	1.76	65	21.00
11	22	1.74	85	28.10
**Average**	23.40 ± 2.00	1.80 ± 0.10	74.50 ± 12.70	23.90 ± 3.10

**Table 2:** Description and units for the outcome measures proposed to measure the performance, physical interaction, and users’ kinematics during trials.

Indicator	Units	Description
Mean Force	[N]	The average value of the resulting force signal $\vec{F}$ acquired during each trial.
Peak Force	[N]	The maximum positive value of $\vec{F}$ during each trial. It describes the initial contact between users and the device, measuring the difficulty to start moving the device.
Mean Torque	[Nm]	The average value of the resulting torque signal $\vec{\tau }$ acquired during each trial. Since the proposed experimental setup considers paths with left and right turnings, this indicator was reported using the modulus or absolute value.
Peak Torque	[Nm]	The highest positive or negative maximum $\vec{\tau }$ value during each trial.
User’s Speed	[m/s]	The average value of the magnitude of the user’s velocity. This indicator was calculated using data of the marker corresponding to the 7th cervical vertebra (C7).
SW Linear Speed	[m/s]	The average value of the magnitude of the smart walker’s linear speed, i.e., the speed in the y-axis direction.
SW Angular Speed	[rad/s]	The average value of the magnitude of the smart walker’s angular speed, i.e., the speed in the y-axis direction.
Cadence	[steps/min]	The total number of full cycles or steps taken within a minute. This indicator was reported as the average cadence during each trial.
Cycle Duration	[s]	The average duration of full gait cycles during each trial.
No. Cycles	-	The total number of cycles or steps taken during each trial.
Hip Flexion ROM	[°]	The average range of motion of the hip flexion angle. Estimated as the average difference between the maximum and minimum angle.
Knee Flexion ROM	[°]	The average range of motion of the knee flexion angle. Estimated as the average difference between the maximum and minimum angle.
Ankle Flexion ROM	[°]	The average range of motion of the ankle flexion angle. Estimated as the average difference between the maximum and minimum angle.
Trial Duration	[s]	The duration of each trial measured in seconds.

**Table 3:** Summary of physical interaction data between users and the AGoRA Smart Walker under several assistance modes.

Indicator	AM	PM	RM	*p*-Value
Mean Force [N]	1.67 ± 0.60	2.22 ± 0.65	5.14 ± 1.53	**2.2 × $10^{-16}$**
Peak Force [N]	4.47 ± 1.23	4.85 ± 0.93 *	11.01 ± 2.35	**1.5 × $10^{-12}$**
Mean Torque [Nm]	0.38 ± 0.13 *	0.38 ± 0.10	0.88 ± 2.35 *	**<2.2 × $10^{-16}$**
Peak Torque [Nm]	2.39 ± 0.68 *	1.56 ±0.40	5.77 ± 0.59 *	**2.2 × $10^{-16}$**

Asterisks mean that the variable is normally distributed. *p*-values in bold indicate significant differences between modes.

**Table 4:** Obtained *p*-values after pairwise comparisons of physical interaction parameters using posthoc tests.

Indicator	AM-PM	AM-RM	PM-RM
Mean Force	**2.2 × $10^{-7}$**	**1.1 × $10^{-12}$**	**2.0 × $10^{-7}$**
Peak Force	**1.9 × $10^{-11}$**	**2.2 × $10^{-16}$**	**3.5 × $10^{-9}$**
Mean Torque	**2.0 × $10^{-16}$**	**1.9 × $10^{-9}$**	**1.1 × $10^{-12}$**
Peak Torque	**2.2 × $10^{-9}$**	**2.0 × $10^{-7}$**	**1.5 × $10^{-16}$**

*p*-values in bold were found to be statistically different.

**Table 5:** Summary of kinematic and additional outcomes during trials.

Indicator	AM	PM	RM	UM	*p*-Value
Users’ Speed [m/s]	0.44 ± 0.05 *	0.46 ± 0.06	0.34 ± 0.04 *	0.77 ± 0.02 *	**2.27 × $10^{-6}$**
SW Linear Speed [m/s]	0.34 ± 0.08	0.33 ± 0.11	0.26 ± 0.05	-	**8.63 × $10^{-9}$**
SW Angular Speed [rad/s]	0.16 ± 0.04	0.12 ± 0.03 *	0.11 ± 0.03	-	**1.31 × $10^{-12}$**
Cadence [steps/min]	51.46 ± 11.73	50.21 ± 10.37	48.61 ± 31.11	53.41 ± 8.36	**4.23 × $10^{-8}$**
Cycle Duration [s]	1.21 ± 0.21 *	1.24 ± 0.23 *	1.48 ± 0.51 *	1.15 ± 0.15	**3.22 × $10^{-7}$**
No. Cycles	6.68 ± 1.75	6.82 ± 1.97	9.32 ± 6.51	4.29 ± 0.67	**6.91 × $10^{-16}$**
Hip Flexion ROM [°]	39.78 ± 4.57 *	43.06 ± 5.93 *	50.84 ± 7.66 *	43.44 ± 4.31 *	**3.83 × $10^{-15}$**
Knee ROM [°]	59.49 ± 6.38	59.34 ± 5.96	58.22 ± 7.25 *	64.28 ± 6.89 *	**1.90 × $10^{-8}$**
Ankle Flexion ROM [°]	27.05 ± 11.57	29.21 ± 5.85	34.90 ± 9.85	28.28 ± 5.16 *	**7.82 × $10^{-7}$**
Trunk Angle [°]	86.35 ± 5.67 *	88.95 ± 4.21	71.39 ± 8.75	85.48 ± 3.96 *	**5.32 × $10^{-5}$**
Trial Duration [s] [°]	13.35 ± 2.18	14.56 ± 6.44	16.78 ± 2.14 *	8.71 ± 1.67 *	**2.0 × $10^{-16}$**

Asterisks mean that the variable is normally distributed. *p*-values in bold indicate significant differences between modes.

**Table 6:** Obtained *p*-values after pairwise comparisons of kinematic and additional parameters using post-hoc tests.

Indicator	AM-PM	AM-RM	AM-UM	PM-RM	PM-UM	RM-UM
Users’ Speed	**3.4 × $10^{-2}$**	**9.2 × $10^{-6}$**	**3.1 × $10^{-10}$**	**3.4 × $10^{-9}$**	**5.9 × $10^{-7}$**	**2.2 × $10^{-15}$**
SW Linear Speed	**1.4 × $10^{-5}$**	**2.3 × $10^{-10}$**	-	**1.4 × $10^{-5}$**	-	-
SW Angular Speed	**6.1 × $10^{-6}$**	**4.1 × $10^{-4}$**	-	**2.2 × $10^{-16}$**	-	-
Cadence	**1.3 × $10^{-5}$**	**4.9 × $10^{-10}$**	**2.0 × $10^{-4}$**	**3.0 × $10^{-3}$**	**5.2 × $10^{-11}$**	**2.1 × $10^{-14}$**
Cycle Duration	**7.7 × $10^{-7}$**	**1.1 × $10^{-13}$**	**7.7 × $10^{-7}$**	**7.7 × $10^{-7}$**	**1.1 × $10^{-13}$**	**2.0 × $10^{-16}$**
No. Cycles	**3.5 × $10^{-2}$**	**3.9 × $10^{-7}$**	**4.5 × $10^{-3}$**	**1.8 × $10^{-10}$**	**1.1 × $10^{-6}$**	**3.8 × $10^{-11}$**
Hip Flexion ROM	5.1 × $10^{-2}$	**2.7 × $10^{-15}$**	**2.0 × $10^{-2}$**	**1.3 × $10^{-8}$**	1.4 × $10^{-1}$	**6.3 × $10^{-8}$**
Knee Flexion ROM	1.9 × $10^{-1}$	**4.0 × $10^{-6}$**	**2.0 × $10^{-16}$**	**1.5 × $10^{-2}$**	**2.0 × $10^{-16}$**	**2.0 × $10^{-16}$**
Ankle Flexion ROM	5.7 × $10^{-1}$	**7.0 × $10^{-7}$**	**4.7 × $10^{-13}$**	**8.6 × $10^{-5}$**	**4.8 × $10^{-2}$**	**4.5 × $10^{-8}$**
Trunk Angle	2.6 × $10^{-1}$	**5.5 × $10^{-6}$**	**5.7 ×** $10^{-2}$	**6.8 × $10^{-7}$**	4.3 × $10^{-1}$	**4.7 × $10^{-4}$**
Trial Duration	**1.5 × $10^{-4}$**	**2.0 × $10^{-8}$**	**5.1 × $10^{-9}$**	**4.3 × $10^{-5}$**	**6.9 × $10^{-15}$**	**2.0 × $10^{-16}$**

*p*-values in bold were found to be statistically different.

## Abbreviations

The following abbreviations are used in this manuscript:

SWs	Smart Walkers
HRI	Human-Robot Interaction
IMU	Inertial Measurement Unit
LiDAR	Light Detection and Ranging Sensor
LRF	Laser Range-Finder
AM	Assistive Mode
RM	Resistive Mode
BMI	Body Mass Index
PM	Passive Mode
AGoRA	Adaptable Robotic Platform for Gait Assistance and Rehabilitation
UM	Unassisted Mode
ROS	Robotic Operating System
BTK	Biomechanical ToolKit Library
ROM	Range of Motion
ANOVA	Analysis of Variance
